# The relationship between fish abundance and benthic community structure on artificial reefs in the Mid-Atlantic Bight, and the importance of sea whip corals *Leptogorgia virgulata*

**DOI:** 10.7717/peerj.7277

**Published:** 2019-07-16

**Authors:** Cara C. Schweitzer, Bradley G. Stevens

**Affiliations:** Department of Natural Sciences, University of Maryland Eastern Shore, Princess Anne, MD, United States of America

**Keywords:** Sea whip, Black sea bass, Biogenic structure, Mid-atlantic bight, Coral, Fish abundance, Temperate reef, Artificial reef

## Abstract

Autogenic engineers (i.e., biogenic structure) add to habitat complexity by altering the environment by their own physical structures. The presence of autogenic engineers is correlated with increases in species abundance and biodiversity. Biogenic structural communities off the coast of Delaware, Maryland, and Virginia (Delmarva) are comprised of multiple species including boring sponge *Cliona celata*, various hydroids (i.e., *Tubularia* sp., *Obelia* sp., *Campanular* sp.), northern stone coral *Astrangia poculata*, sea whips *Leptogorgia virgulata*, and blue mussels *Mytilus edulis.* Sea whips are soft corals that provide the majority of vertical height to benthic structure off the coast of the Delmarva peninsula. The mid-Atlantic bight is inhabited by several economically valuable fishes; however, data regarding habitat composition, habitat quality, and fish abundance are scarce. We collected quadrat and sea whip images from 12 artificial reef sites (i.e., shipwrecks) ranging from 10 to 24 m depth to determine proportional coverage of biogenic structures and to assess habitat health, respectively. Underwater video surveys were used to estimate fish abundances on the 12 study sites and determine if fish abundance was related to biogenic coverage and habitat health. Our results showed that higher fish abundance was significantly correlated with higher proportional sea whip coral coverage, but showed no significant relationship to other biogenic structure. Assessment of sea whip condition (as a damage index) showed that sea whip corals on artificial reefs off the Delmarva coast exhibited minor signs of degradation that did not differ significantly among study sites.

## Introduction

Structurally complex habitats, such as cobble and rock reefs, and natural or artificial reefs, are profoundly important for fish and crustaceans by providing spatial refuge and feeding sites (Robertson & Sheldon, 1979; Hixon & Beets, 1993; Forrester & Steele, 2004; Scharf, Manderson & Fabrizio, 2006; Johnson, 2007; Cheminee et al., 2016; Gregor & Anderson, 2016). Structural habitat can be essential for the settlement and proliferation of autogenic engineers (e.g., corals, sponges, bivalves, sea grasses). The presence of biogenic structure can increase the quality of habitats and can affect habitat selection, abundance of economically valuable species, and survival and settlement of fishes (Gibson, 1994; Garpe & Öhman, 2003; Diaz, Solan & Valente, 2004; Miller et al., 2012; Komyakova, Jones & Munday, 2018; Seemann et al., 2018; Soler-Hurtado, Megina & López-González, 2018). This is most evident when biogenic structures are damaged or undergo mortality events, which often results in regional loss of fish biomass, biodiversity, and abundance (Jones et al., 2004; Lotze et al., 2006; Thrush et al., 2008; Dudgeon et al., 2010; McCauley et al., 2015). The extent to which autogenic engineers influence fish abundance has been well studied in tropical marine ecosystems (Richmond, 1996; Downs et al., 2005; Hughes et al., 2010; Newman et al., 2006), but is poorly understood within temperate rock reef systems of the Mid-Atlantic.

Within the Mid-Atlantic Bight, biogenic structure primarily consists of boring sponge *Cliona celata*, various hydroids (i.e., *Tubularia* sp., *Obelia* sp., *Campanular* sp.), northern stone coral *Astrangia poculata,* sea whip corals *Leptogorgia virgulata* (Gotelli, 1991; Guida et al., 2017), and blue mussels *Mytilus edulis* (Steimle & Zetlin, 2000; Cullen & Stevens, 2017). Among this community, sea whip corals are the primary contributors of additional height to artificial and natural rock reefs. Previous studies conducted within coral reef ecosystems have demonstrated that rugosity and coral height are the strongest predictors of fish biomass (Harborne, Mumby & Ferrari, 2012). Benthic rock reefs and artificial reefs within the Mid-Atlantic Bight are poorly studied and the composition of benthic biogenic structures is unknown.

Marine benthic structure within the Delaware, Maryland, Virginia peninsula (Delmarva) portion of the Mid-Atlantic Bight consists of both natural rock reefs and artificial reefs. Natural rock reefs are composed of rock, mud, and clay outcrops, and artificial reefs, both unintentional (e.g., shipwrecks) and intentional (e.g., concrete blocks and pipes, subway cars, ships). Natural rock reefs are sparse, sporadically distributed and highly fragmented. Artificial reefs provide the dominant source of benthic structure either through accidental shipwrecks or constructed through artificial reef programs. Artificial reef construction has become a popular way to increase regional habitat production, biodiversity, fish abundance, and to restore biogenic structure (Bohnsack, 1989; Grossman, Jones & Seaman, 1997; Sherman, Gilliam & Spieler, 2002; Granneman & Steele, 2015; Scott et al., 2015; Smith et al., 2017). Artificial reef sites are constructed regularly off the coast of the Delmarva peninsula, with the goal of increasing the abundance of structure-oriented fish of economic value. Some of these species, such as black sea bass *Centropristis striata*, and tautog *Tautoga onitis,* reside directly within the structures; whereas others, such as Atlantic croaker *Micropogonias undulatus*, and summer flounder *Paralichthys dentatus*, are commonly found on sandy bottoms near benthic structures as adults (Feigenbaum et al., 1989; Hostetter & Munroe, 1993; Scharf, Manderson & Fabrizio, 2006; Fabrizio, Manderson & Pessutti, 2013)*.*

Habitat quality on benthic rock reefs and its relationship to species abundance has been largely neglected in the Mid-Atlantic Bight. Previous research investigating habitat association for economically important species (e.g., black sea bass) within the Mid-Atlantic Bight focused on benthic hardness and did not consider biogenic composition (Fabrizio, Manderson & Pessutti, 2013). Diaz, Solan & Valente (2004) performed a small-scale study investigating fish abundance in relation to biogenic structure in relation to density of patches of infaunal tubes at Fenwick Shoals off the coast of Delaware. They found that patch size and presence of biogenic structure was significantly related to juvenile fish abundance for that site. However, there are still insufficient data to suggest that these results are representative of habitat patches throughout the Mid-Atlantic Bight. To date, there is a paucity of data regarding the composition variability and degree of coverage of biogenic structure on natural or artificial reefs, and its relationship to fish abundance within the Mid-Atlantic Bight.

It is important to understand how sensitive, stable, or resilient complex habitats are in order to preserve the sustainability of economically valuable species. An improved understanding in the relationship between benthic habitat quality and fish abundance in the Mid-Atlantic Bight can lead to improvements in management policies. Assessments in habitat quality is commonly achieved by monitoring indicator species that are selected based on their sensitivity to habitat disturbances, and can be effective in the evaluation of an ecosystems response to stressors (Andersen, 1986; Simberloff, 1998; Siddig et al., 2016). Since sea whip corals primarily contribute to additional vertical relief, they may be more susceptible to fishing disturbances, and external damage and over-colonization can be easily quantified, we hypothesize that sea whip corals may be an indicator species for benthic habitats in the Mid-Atlantic Bight.

We undertook a study to determine the structure of marine biogenic communities and their relationship to fish abundance in the Delmarva portion of the Mid-Atlantic Bight. Our study had four specific objectives which were: (1) to determine the species composition and proportional coverage of biogenic structure at various artificial reefs; (2) to estimate relative fish abundance at those sites; (3) to estimate habitat quality using a damage index (DI) for sea whips; and (4) to determine the relationships between fish abundance and the quantity and quality of biogenic habitat.

## Methods

### Description of study sites

**Figure 1:**
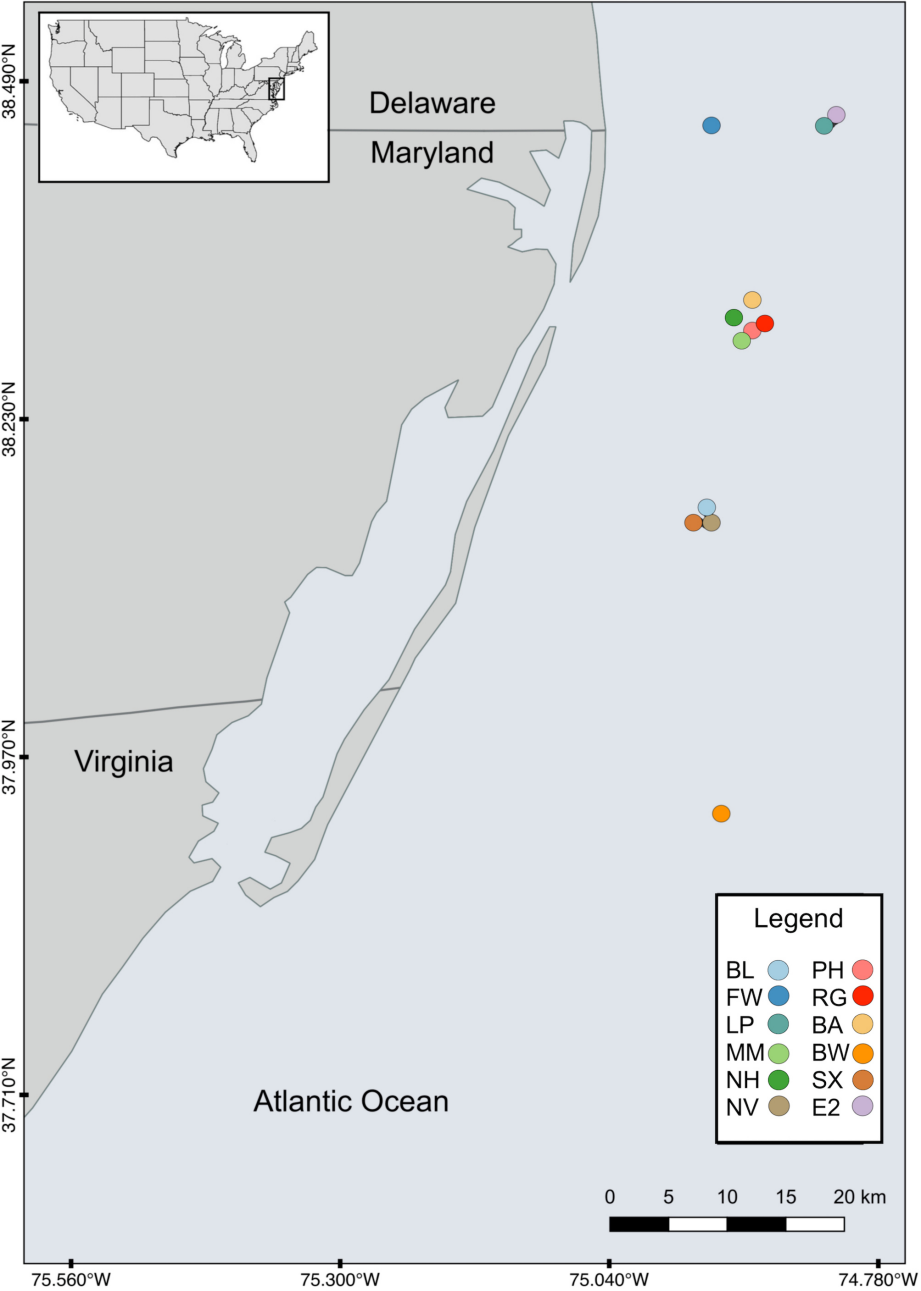
Map of study sites. Map of the study area showing the locations of the 12 artificial reefs off the coast of the Delmarva (Delaware, Maryland, Virginia) peninsula.

Twelve artificial reef study sites were selected based on site age and SCUBA accessibility (Table 1). Sites were located off the cost of the Delaware, Maryland, and Virginia (Delmarva) peninsula between the latitudes of 37°N and 38.5°N ranging from 9 to 32 km off the coast (Fig. 1) at depths from ∼10 to ∼24 m. The maximum distance between sites (Site FW and RG) was ∼60.1 km and the minimum separation distance (between Sites EP and E2) was ∼0.52 km. The majority of the sites (*n* = 8) were intentionally sunk in association with the Maryland Artificial Reef Program, and the remaining four were natural wrecks. Both sites PH and RG became separated into two sections with approximately 122 m and 27 m between each section, respectively.

**Table 1:** Table showing approximate age, name abbreviations, and depth of the study sites. Month surveyed is the month fish abundance surveys were completed. Survey method states whether the fish abundance survey was conducted via line transect method (Line) or via stationary camera method (Stationary). Category states whether sites were constructed or naturally sank. Site names are common names of the wreck or region; however; some site names are not universal.

Site	Site name	Abbr.	Approx. age (y)	Approx. depth (m)	Month surveyed	Survey method	Category
1	Fenwick Shoals	FW	120	10	July	Line	Unintentional
2	Elizabeth Palmer	EP	104	23.5	July	Stationary	Unintentional
3	EP2	E2	100	24	July	Stationary	Unintentional
4	Pharoby	PH	37	20	June	Stationary	Deliberate
5	Blenny	BL	30	23.5	July	Line	Deliberate
6	Kathleen Riggins	RG	28	16.5	June	Stationary	Unintentional
7	Memorial Barge	MM	26	18	—	—	Deliberate
8	Sussex	SX	24	24	July	Line	Deliberate
9	Navy Barge	NV	19	20	July	Line	Deliberate
10	New Hope	NH	2	19	August	Line	Deliberate
11	Barge	BA	0.5	18	August	Line	Deliberate
12	Boiler Wreck	BW	NA	24	July	Line	Deliberate

There have been few studies on habitats in the Mid-Atlantic Bight by SCUBA or other in-situ methods due to unpredictable weather and turbid conditions. For these reasons, diving and data collection were restricted to the months of June through November during 2017 and 2018 and only conducted on days with a wave height ≤ one m. Fish abundance surveys were conducted June through August. If quadrat sampling could not be completed during the same sampling day, the site was resampled at a later time. Bottom temperatures were collected via a Castaway^®^ CTD (Sontek Inc., San Diego, CA, USA) once daily. During these months bottom water temperatures ranged from 9.31 °C to 22.57 °C and surface temperatures ranged from 13.48 °C to 27.23 °C. Bottom visibility ranged from ∼0.5 to ∼18 m. Neither quadrat nor video data could be collected on days with bottom visibility <1.5 m.

### Data collection

Quadrat sampling was used to estimate the proportional coverage of the dominant biogenic organisms: boring sponge, *C. celata,* various hydroid species (e.g., *Tubularia sp., Obelia sp., Campanularia sp.*)*,* northern stone coral *A. poculata,* sea whip corals *L. virgulata,* and blue mussels *M. edulis*. Quadrat images (*n* = 11 to 60) were taken by SCUBA divers with a Canon DSLR camera in a housing attached to a 0.25 m^2^ PVC frame.^1^ 1Reference to trade names does not imply endorsement by either the University of Maryland Eastern Shore or funding sources.Images were taken at one m intervals along the long axis of the artificial reef for 30 m, or to end of the wreck. Quadrat sampling was conducted once at each of the sites.

To assess sea whip damage, images of sea whips were taken with GoPro^®^ *Hero* 4 action camera.^1^ If sea whips were abundant (e.g., >1 m^−2^), a subset of sea whips was haphazardly selected and photographed. However, if sea whip abundance was low, then all sea whips present were photographed.

To estimate fish abundance on artificial reefs, an underwater video survey was conducted via two methods: (1) line transect method, and (2) stationary cameras (Table 1). Line transects were conducted at eight sites while stationary camera surveys were conducted at four of sites. The latter method was used primarily to estimate fish abundance for an artificial reef building project and incorporated into this analysis. Line transects were conducted for 30 m along the long axis of the site, or until the end of the wreck. Divers swam along the transect approximately one m above the wreck with the camera facing at a slight angle toward the wreck surface. The mean duration of line transect videos was 306 s ± 102 s SD. Stationary surveys were conducted by placing non-baited aluminum tripods, each of which bore two GoPro^®^ camera placed at 90° angles. Two tripods were placed facing the wreck at a distance of approximately one m from where fish were observed. At Sites 3 and 4, tripods were also placed in the open bottom area between each section to determine the abundance and behavior of fish at those sites. Cameras were left on tripods to record for 45 min, and then retrieved. Stationary camera observations were repeated at least twice at each location, but line transect counts were not repeated due to hazardous weather that restricted diving frequency.

### Data analysis

Images were analyzed with image analysis software ImageJ (version 2.0.0-rc-69/1.52J, NIH) and statistical analysis was completed with R statistical software (v 3.5.2; R Core Team, 2018). Proportional cover for each of the biogenic species was estimated by outlining regions of interest (ROI) in each quadrat image. For sea whip corals, ROI were drawn over the projection of the sea whip on the surface. Analysis of similarities (ANOSIM) based on the Euclidean distance metric was used to test for differences between biogenic structure assemblages at sampling sites. A non-metric multidimensional scaling (NMDS) was used to visualize similarities and differences in biogenic composition throughout the research sites. Five biogenic species composed of two corals and three non-coral organisms were included in the NMDS analysis, which represent the dominant structural organisms that inhabit the Mid-Atlantic.

Due to the frequency of low bottom visibility during video surveys, identification of species was substantially impaired, such that only fish relatively close to the camera could be identified. Therefore, fish abundance was estimated for all fish present and not separated by species. To estimate fish abundance on artificial reefs, we used a modified method of the fish MeanCount method, which is defined as the mean number of individuals observed in a series of frames throughout a viewing interval (Bacheler & Shertzer, 2014). To maintain independence between frames and statistical independence, 12 frames were randomly selected from the line transect surveys and the number of fish within each frame were counted. The MeanCount was calculated as the mean of those 12 frame counts. Since the stationary camera surveys produced two videos, the 12 randomly selected frame counts for each video were averaged per site. In addition to MeanCount, the highest number of fish observed in a single frame during the video (MaxNo) was also reported. Relationships between fish abundance (MeanCount) and coverage of biogenic structure and at each site were analyzed with a linear model (LM) with fish abundance (i.e., MeanCount) as the response variable and biogenic structure species as the predictors. A logit transformation was applied to all proportional data before LM analysis.

To estimate sea whip damage, we initially calculated the relative area of sea whips in using each image using ROIs via a line segment tool that was set at the same width as the sea whip branches. We then calculated the damaged area or region of overgrowth as a proportion of total line length. Proportional damage of individual sea whip corals was averaged at each site, and the mean value was used to assign a damage index (DI) from 1 to 5 (Table 2) as described in Schweitzer, Lipcius & Stevens (2018). The proportional data was analyzed with a LM to determine if there was a was a difference in sea whip DI between sites and if DI had an effect on fish abundance.

**Table 2:** Damage index classifications. Criteria used to classify individual sea whip damage index (DI) and overall habitat DI for images captured. For individual sea whips, damage is defined as any visible tissue damage, exposed skeletal structure, or overgrowth by hydroids or bryozoans.

DI	Damage	Description
1	Minimal	<0.05 damage or overgrowth
2	Minor	0.06–0.25 damage or overgrowth
3	Moderate	0.26–0.50 damage or overgrowth
4	Severe	0.51–0.75 damage or overgrowth
5	Critical	>0.75 damage or overgrowth

## Results

### Composition of artificial reefs

**Figure 2:**
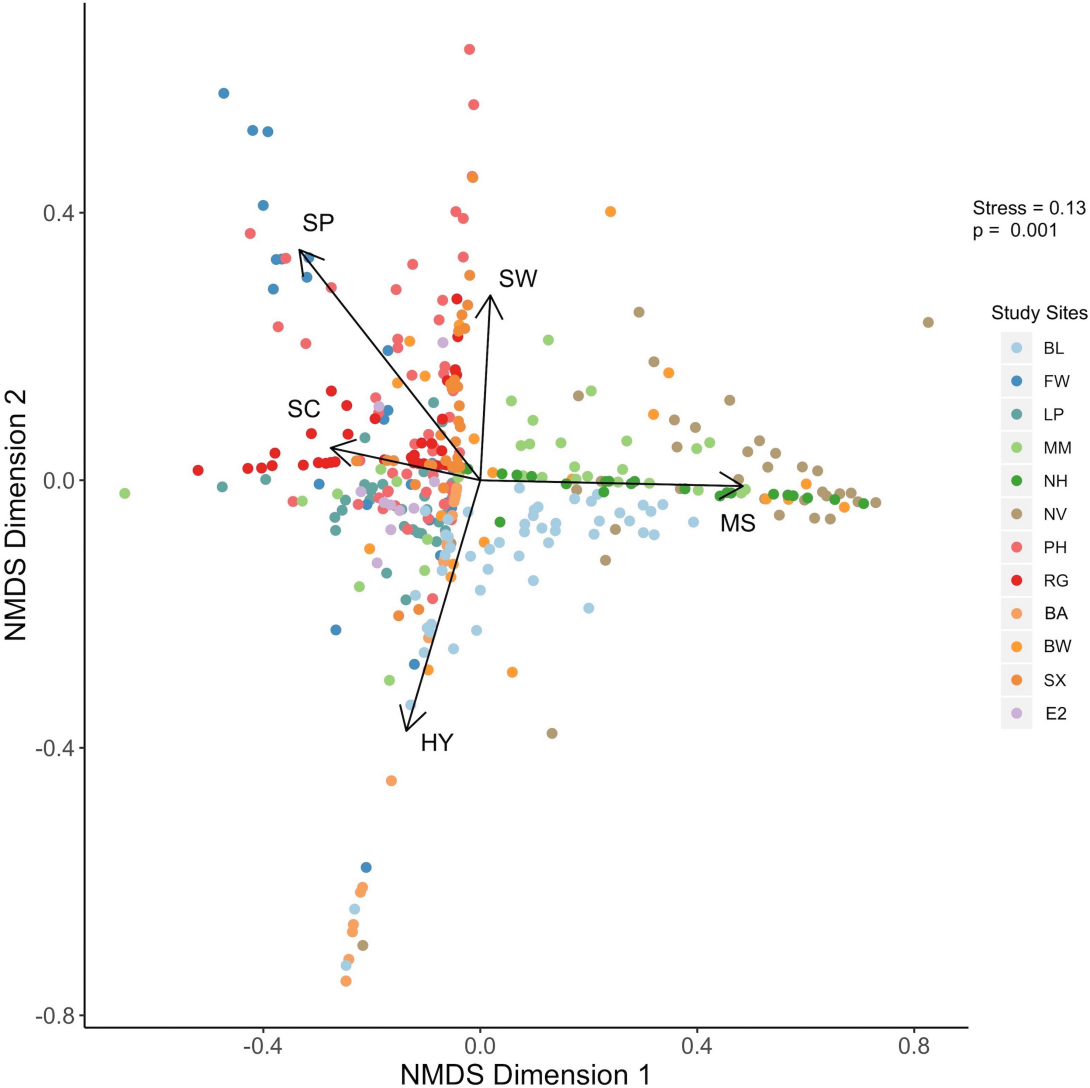
NMDS plot. Results of the nonmetric multidimensional scaling analysis depicting biogenic structure assemblages on the 12 artificial reef sites off the Delmarva coast. *P* value is from the ANOSIM analysis. Variables: SW, sea whip corals; SC, northern stone coral; SP, boring sponge; MS, blue mussel; HY, hydroids.

**Figure 3:**
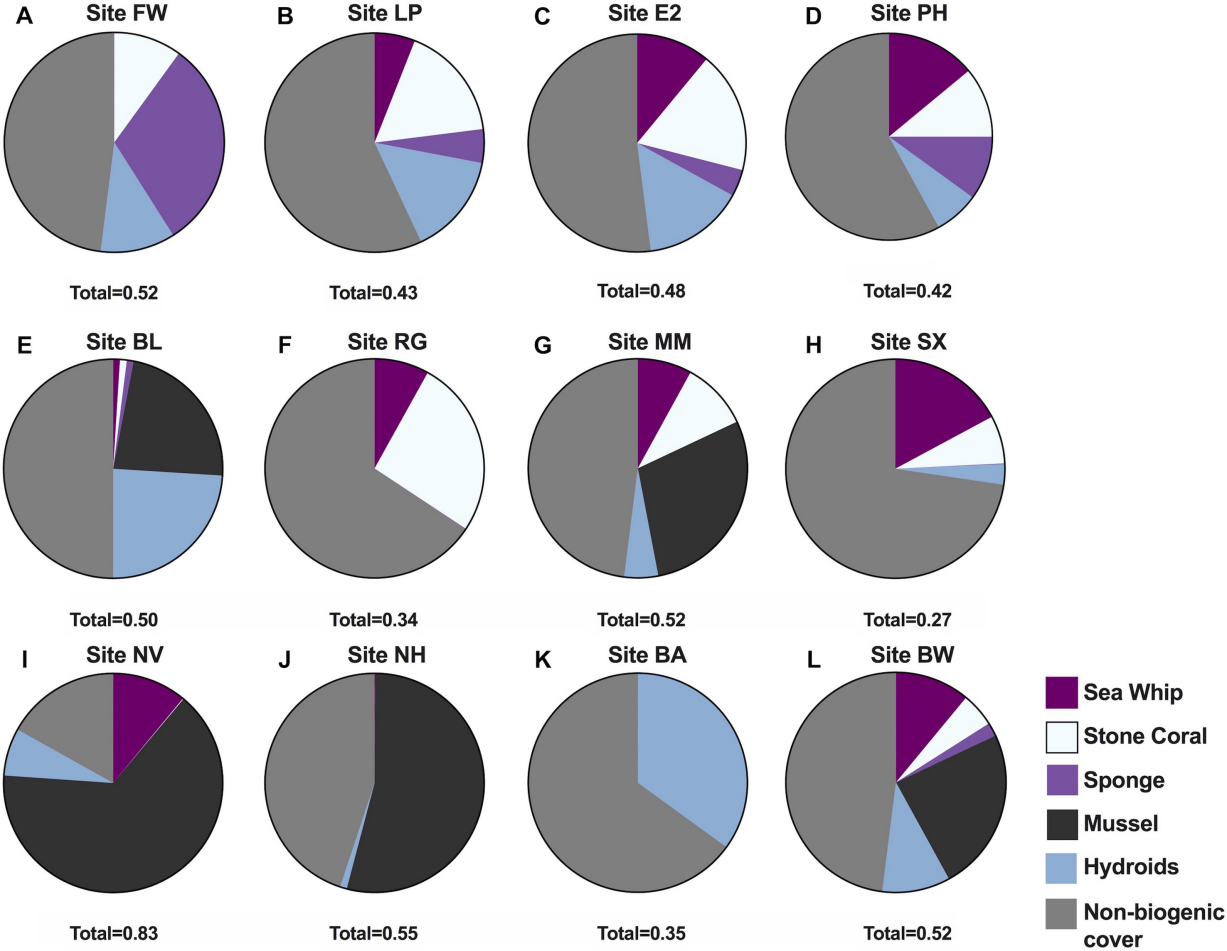
Proportional cover of five structure-forming species groups at the 12 study sites. Pie charts showing the proportions of the five biogenic structures at the 12 study sites (A) Site FW, (B) Site LP, (C) Site E2, (D) Site PH, (E) Site BL, (F) Site RG, (G) Site MM, (H) Site SX, (I) Site NV, (J) Site NH, (K) Site BA, (L) Site BW. Total, Cumulative total coverage of all five biogenic species groups from quadrat images.

Data derived from quadrat images showed a significant difference, but with some overlap in biogenic assemblages between study sites (ANOSIM *R* = 0.32; *p* = 0.001; Fig. 2). The mean proportional coverage of biogenic structure on artificial reefs off the Delmarva coast was 0.47 ± 0.14 (mean ± SD). Proportional coverage was lowest at Site SX (0.27), and greatest at Site NV (0.83; Fig. 3). Sea whip corals *L. virgulata* and northern stone coral *A. poculata* were present on 10 of the 12 sites. One of the two sites void of sea whip corals was constructed 6 mo prior to the quadrat survey and only exhibited colonization by hydroid species (Site BA; Table 3). Blue mussel *M. edulis* beds were found on 5 of the 12 sites. Boring sponge *C. celata* was observed at eight of the 12 sites. Site BW was the only location that contained all five structure-forming species.

Results from the NMDS supported the results from the ANOSIM such that some sites showed distinction in biogenic structure communities, while some sites showed considerable overlap (Fig. 2). Northern stone coral and blue mussels were negatively correlated. Sites NV and NH were associated with blue mussel coverage, while sites EP, E2, PH, and RG were associated with northern stone coral (Fig. 2). Sea whip corals and hydroids were negatively correlated. Sites PH, RG, MM, SX, NV, and BW were associated with sea whips. Sites BL and BA were associated with hydroids. Sites FW, and PH were associated with the boring sponge (Fig. 2).

**Figure 4:**
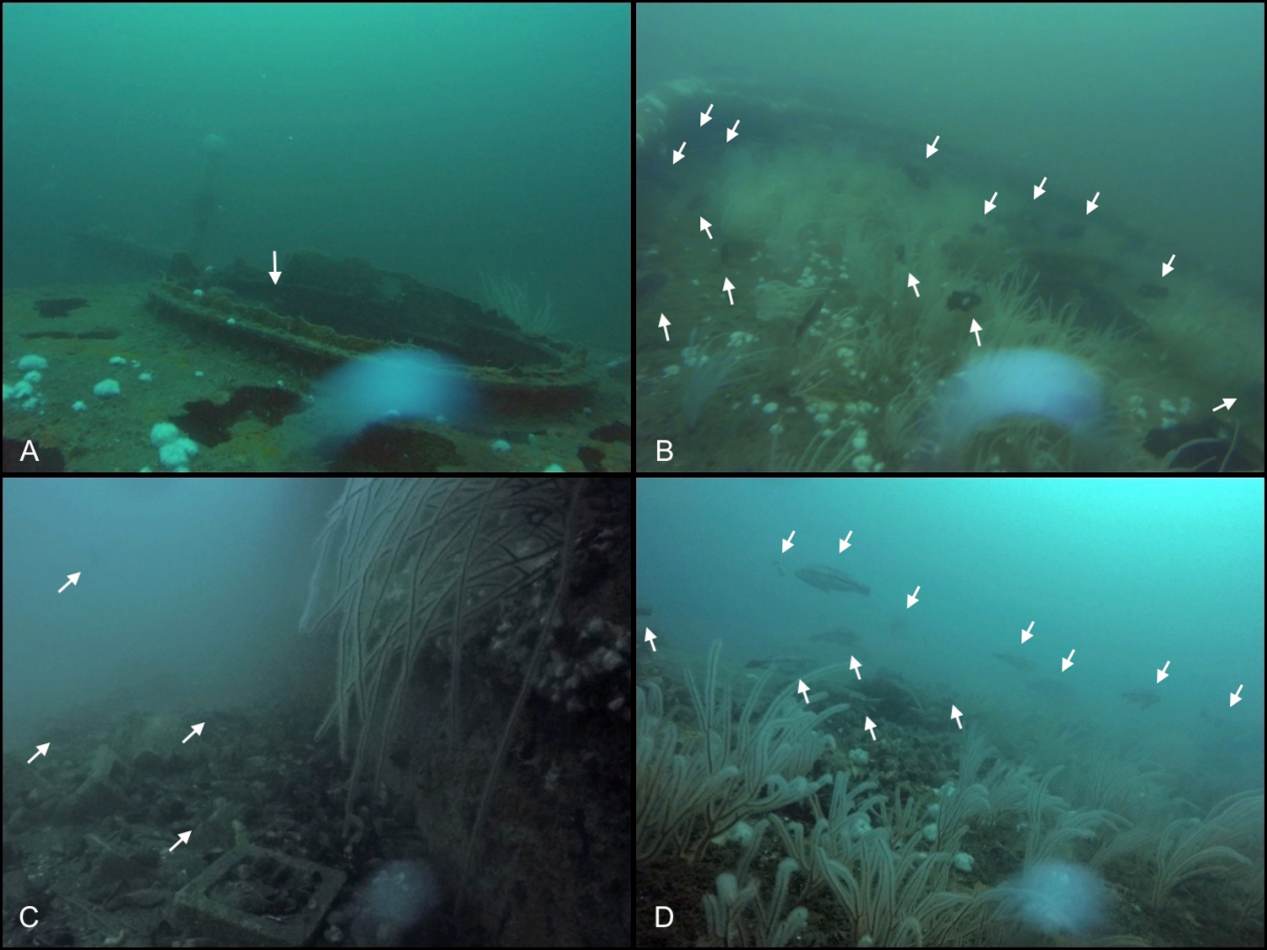
Comparisons of fish aggregations on sites. Photos taken at two locations at Sites SX and NV. (A) Region of Site SX with minimal biostructure. White arrow highlights the single fish located within this frame. (B) Photo taken during the same dive survey as 4A. Region of Site SX with increased sea whip coverage. White arrows show the location of the 14 fish seen within this frame. (C) An area of Site NV that is mostly composed of rock, broken shells and concrete blocks with a single sea whip coral and some colonies of northern stone coral on the wall of the wreck. White arrows show the locations of the 4 fish in the frame. (D) Photo taken during the same dive survey as 4C showing an area with increased sea whip colonies. White arrows show the location of the 12 fish within the frame. Photo credit: Cara C. Schweitzer.

**Figure 5:**
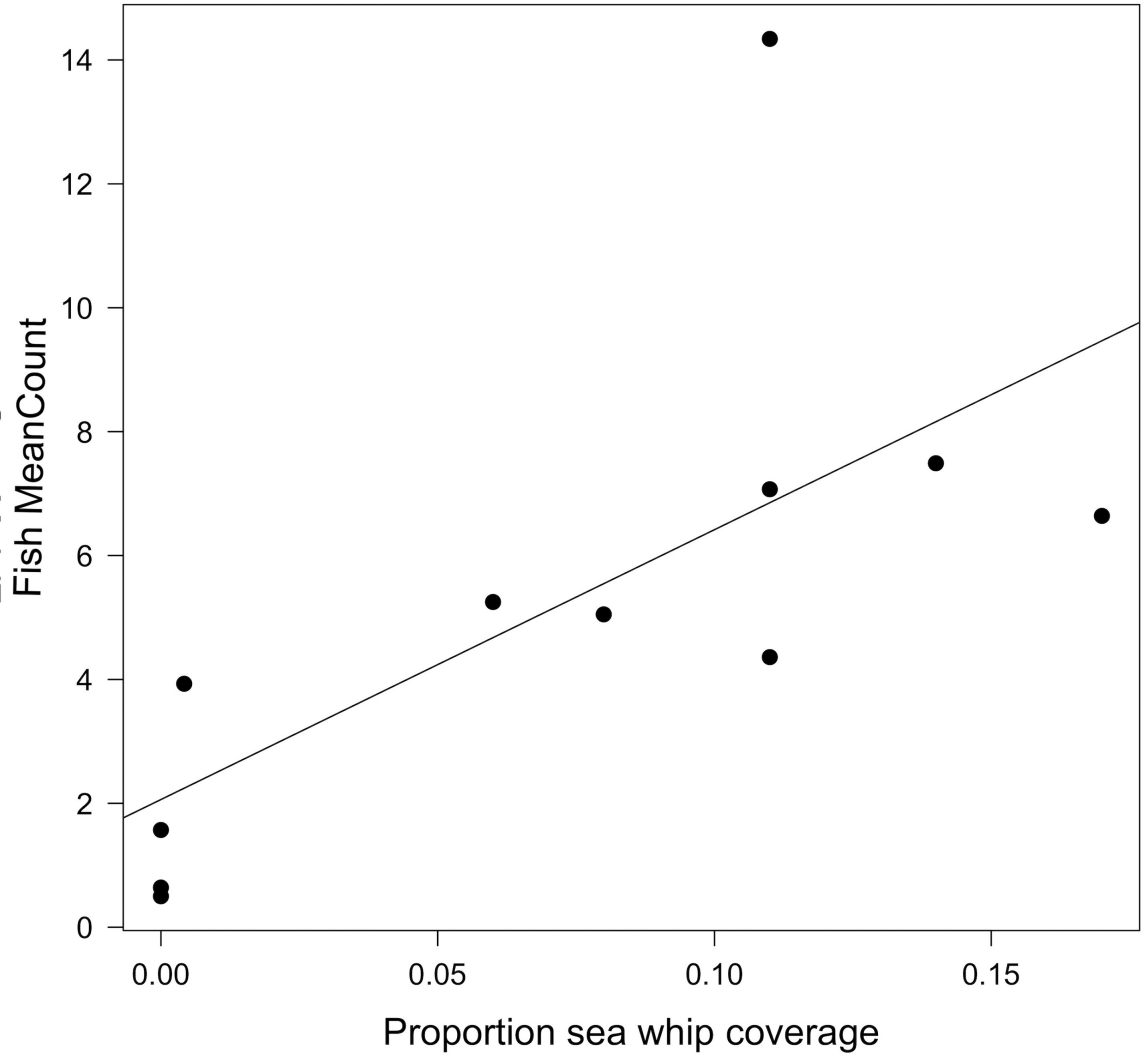
Relationship between fish MeanCount and proportional sea whip coverage. Linear model showing the relationship between fish MeanCount and proportional sea whip coral coverage for 11 of the 12 study sites. There is a significant positive correlation (*p* = 0.018; *r*^2^ = 0.48).

Fish MeanCounts were obtained from 11 of the 12 sites. Visibility was too poor for a video survey to be conducted at Site MM, and hazardous weather prevented additional outings. The highest fish count observed (MaxNo; Table 4) in the video survey was also reported because fish were often observed aggregated near biogenic structure, specifically sea whip corals, rather than dispersed throughout the wreck (Fig. 4). The two highest MeanCounts were at Sites E2 and PH, while the lowest were at Sites FW and NH, whereas the Sites E2 and SX had the highest MaxNo (Table 4). No fish were observed swimming on open sandy bottom. Since MeanCounts and MaxNo were highly correlated (*r*^2^ = 0.94) results are reported in MeanCounts. A LM analysis of all 12 sites showed that total proportional coverage of biogenic structure was not significant predictor to fish abundance (ANOVA, *F* = 0.14; *p* = 0.72; *r*^2^ = 0.02). Proportional sea whip coverage, however, was the only significant predictor to fish MeanCounts (*p* = 0.028; *r*^2^ = 0.48; Table 5; Fig. 5). An additional analysis was conducted for sites where video surveys were conducted via line transect method to determine if stationary cameras created an upward bias. Similarly, line transects showed a significant relationship between MeanCount and proportional sea whip coral coverage (*p* = 0.014; *r*^2^ = 0.69).

**Table 3:** Summary table of quadrats. Summary table of proportional cover of biogenic structures by site. *n* quadrats are the number of images taken and analyzed at each site. $\overline{x}$ is the mean proportional coverage for each variable: SW, sea whip coral; SC, northern stone coral; SP, boring sponge; MS, blue mussel; HY, hydroids.

Site	*n* quadrats	$\overline{x}$ SW	$\overline{x}$SC	$\overline{x}$ SP	$\overline{x}$MS	$\overline{x}$ HY
FW	27	0.00	0.10	0.31	0.00	0.11
LP	36	0.06	0.17	0.05	0.00	0.15
E2	11	0.11	0.18	0.04	0.00	0.15
PH	60	0.14	0.11	0.10	0.00	0.07
BL	51	0.01	0.01	<0.01	0.23	0.24
RG	41	0.08	0.26	<0.01	0.00	0.00
MM	33	0.08	0.10	0.00	0.29	0.05
SX	31	0.17	0.07	<0.01	0.00	0.03
NV	37	0.11	<0.01	0.00	0.65	0.07
NH	27	<0.01	0.00	0.00	0.54	0.01
BA	31	0.00	0.00	0.00	0.00	0.35
BW	27	0.11	0.05	0.02	0.24	0.1

**Figure 6:**
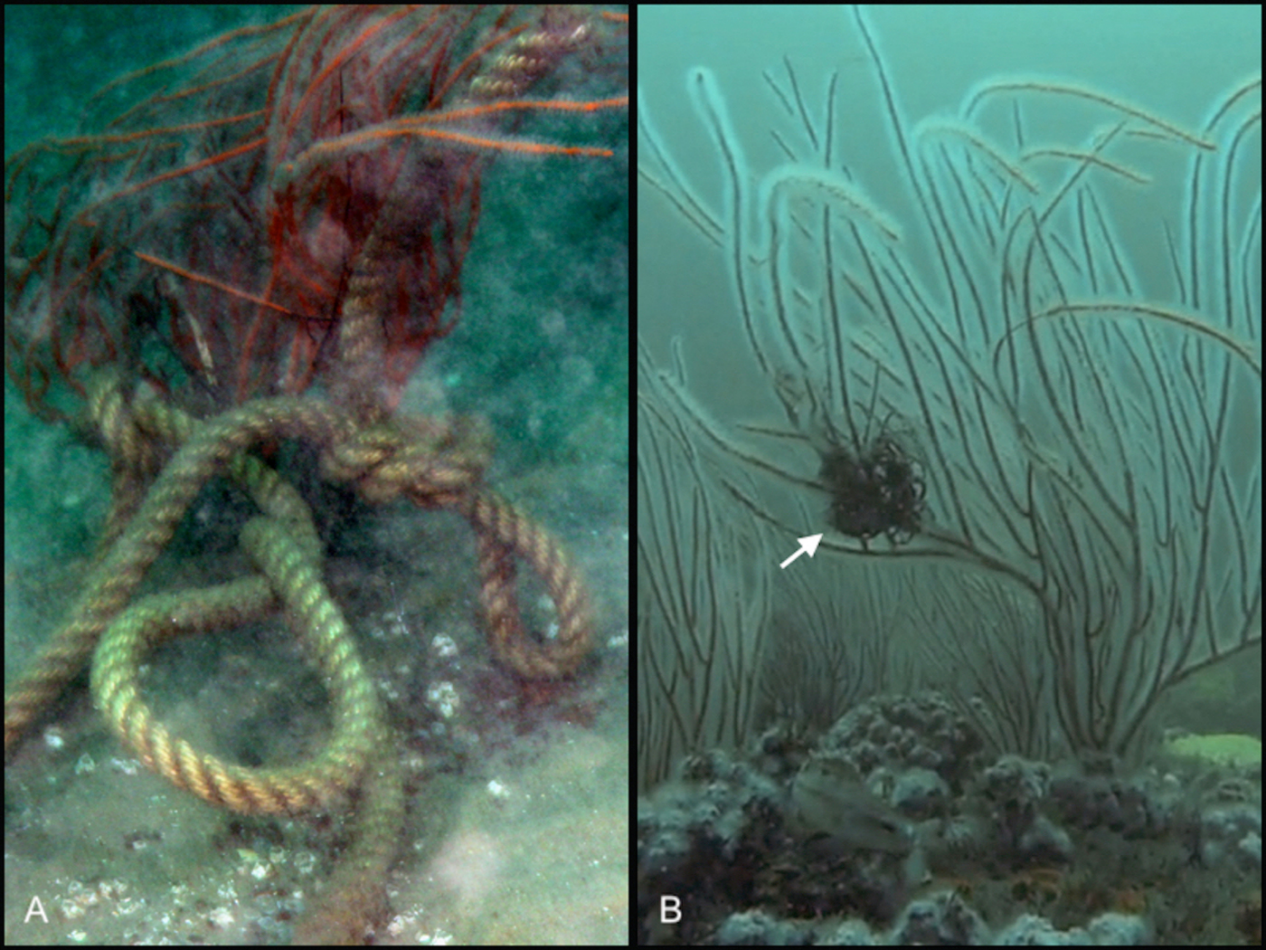
Anthropogenic disturbance effecting sea whip corals. Two photographs showing representative examples of anthropogenic disturbance observed at the research sites. (A) Sea whip coral from Site PH entangled in rope. (B) Sea whip from Site NV with fish line entangled around a portion of branches. Photo credit: Cara C. Schweitzer.

**Figure 7:**
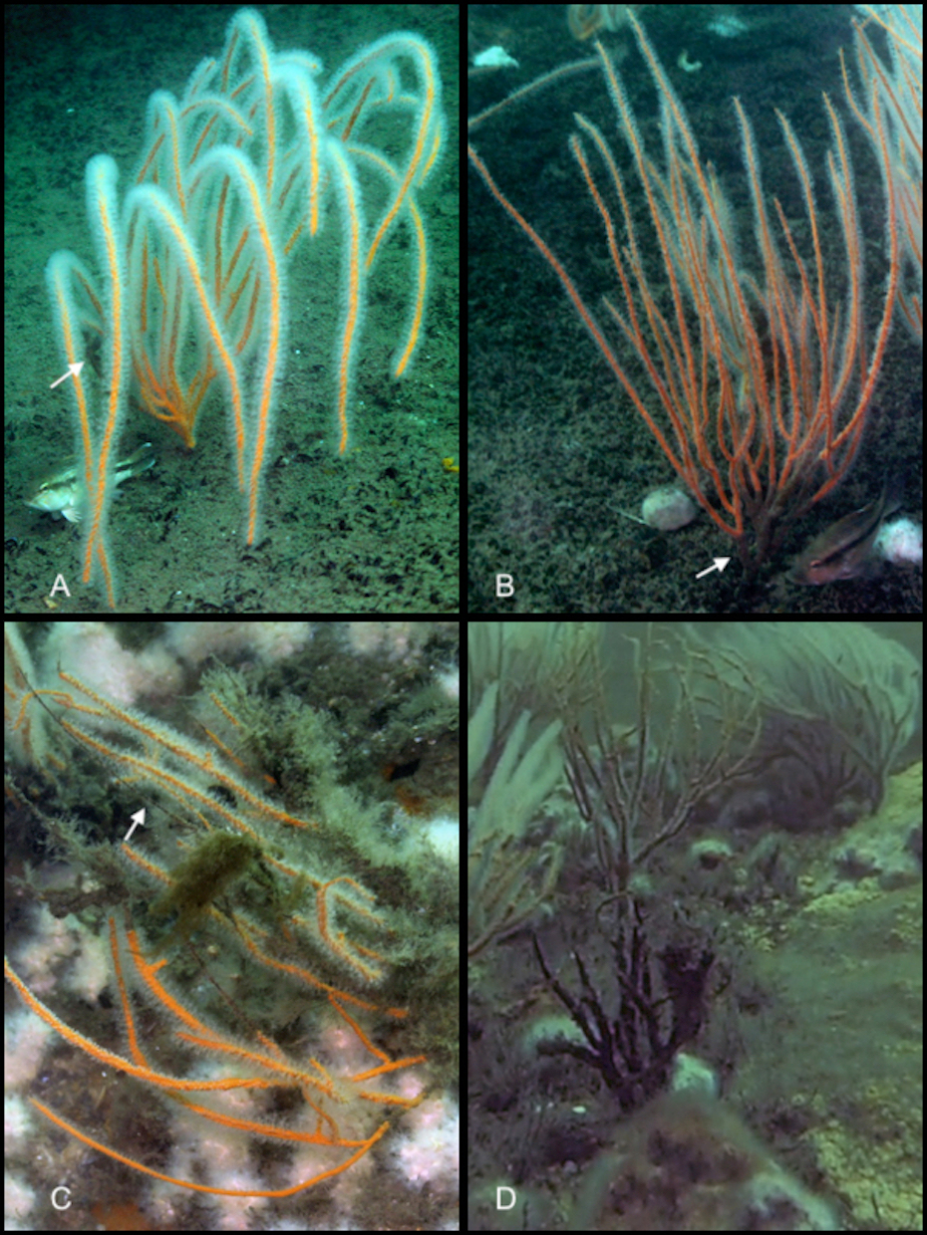
Sea whip corals at various levels of damage. Photographs showing sea whip corals with four different degrees of damage. (A) Sea whip coral exhibiting a minimal proportional damage index of 0.02. White arrow highlights the region of damage. (B) Sea whip coral exhibiting minor proportional damage index of 0.13, localized at the base of the coral. (C) Sea whip coral exhibiting a severe proportional damage index of 0.51. The white arrow is showing a region where the tissue has completely decayed, exposing the skeletal structure. This coral also exhibits colonization of hydroids. (D) Sea whip coral exhibiting critical proportional damage index of 1.00 with no live tissue remaining. Photo credit: Cara C. Schweitzer.

Evidence of habitat disturbance due to fishing (e.g., lures, fishing line, abandoned traps) was observed at 10 of the 12 sites (all but Sites E2 and BA). Observations of tangled fishing line were common at edges of shipwrecks. Fishing gear was observed in direct contact with sea whips corals at nine of the 10 sites where sea whips occurred (Fig. 6). To determine if cumulative sea whip damage was related to reduced fish abundance we analyzed a total of 193 sea whip images from 10 of the 12 study sites, excluding Sites FW and BA, where sea whip corals were absent. Sea whips at most sites exhibited various levels of degradation (Fig. 7). However, despite evidence of fishing disturbance at all sites, with the exception of Site E2, the mean damage index (DI) was 0.15 ± 0.19 SD for all sites, which is indicative of minor levels of degradation (Table 6). Site LP showed the highest DI with a mean of 0.26 ± 0.19 indicating a moderate level of degradation, however this was not significantly different from the other sites (ANOVA; *p* = 0.061) therefore no further analysis was conducted. Consequently, we could not determine if sea whip corals could serve as an indicator species for habitat quality.

**Table 4:** Table showing fish MeanCount and MaxNo. Summary of fish MeanCount and MaxNo for underwater video census surveys.

**Site**	**MeanCount**	**SD**	**MaxNO**
FW	0.50	0.76	2
EP	5.25	2.18	14
E2	14.4	5.67	35
PH	7.49	3.07	24
BL	3.93	5.44	18
RG	5.05	2.66	18
MM	–	–	–
SX	6.64	7.56	27
NV	4.36	4.86	15
NH	0.64	2.41	3
BA	1.57	0.84	7
BW	7.07	4.92	19

## Discussion

The presence of autogenic engineers often increases habitat quality resulting in increases in species abundance and biodiversity across terrestrial, freshwater, and marine ecosystems (Jones, Lawton & Shachak, 1994; Hastings et al., 2007), however, not all can be considered equivalent having positive correlations with species biodiversity and abundance (Jones, Lawton & Shachak, 1997; Daleo et al., 2004). Therefore, it is important to understand the relationships between composition of biogenic structure and fish abundance. Benthic rock reefs and artificial reefs within the Mid-Atlantic Bight are poorly studied and inhabited by multiple economically important species (Hostetter & Munroe, 1993; Shepherd, Moore & Seagraves, 2002) that are exploited both recreationally and commercially. Many of these species (e.g., black sea bass and tautog) are considered structure oriented, but it remains unclear if biogenic structure affects their habitat selection. Insights into the relationships between biogenic structure and fish abundance will be useful for developing ecosystem-based fisheries management (EBFM).

**Table 5:** Results from linear model of fish MeanCount vs proportional cover. ANOVA results from the linear model analysis for 11 of the 12 sites. Fish MeanCount is the response variable and biogenic structural species are the predictor variables.

**Variable**	**Sum Sq**	*F* value	*df*	*P***value**
Sea whips	73.87	9.31	10	0.028
Stone Coral	20.01	2.52	10	0.173
Sponge	0.13	0.17	10	0.904
Blue mussel	7.87	0.99	10	0.365
Hydroids	11.21	1.41	10	0.288

In this study we measured habitat composition and relative fish abundance on 12 artificial reef sites to determine if relationships existed between biogenic structure and habitat use by fish. We concluded that fish abundance was significantly correlated with sea whip coral as a proportion of total cover and that fish were often aggregated near sea whips. In fact, sites without sea whips, or having a proportional abundance of <0.01, had low values for both fish MeanCounts and MaxNo. Within the Mid-Atlantic Bight, sea whip corals are the primary autogenic engineer that contributes to height of benthic structure, increasing the structural complexity of such habitats. In previous studies, coral height has been found to be a significant predictor of fish abundance and biodiversity within coral reef systems (Hoyle & Harborne, 2005). Due to their height, sea whip corals can be susceptible to disturbance (e.g., fishing) that may result in damage and degradation (Schweitzer, Lipcius & Stevens, 2018), which could lead to reduced fish abundance.

Habitat degradation is commonly correlated with a reduction in biodiversity and abundance of associated species (Wilson et al., 2006). We observed sea whips entangled in fishing line and rope, along with various levels of damage to colonies throughout the study sites. However, our study sites did not differ significantly from each other; therefore, we could not determine the effect of sea whip damage on fish abundance. This result is not surprising because, these sites are exposed to seasonal recreational fishing pressure, and are seldom fished by commercial fishers. Seasonal fishing pressure (i.e., June through September) may allow for some recovery from disturbance, however sea whip recovery rate from tissue damage is poorly understood. Previous studies have shown that commercial traps drag along the ocean bottom upon retrieval, running over and breaking sea whips (Schweitzer, Lipcius & Stevens, 2018), which may accelerate degradation. In order to test the hypothesis that sea whip coral health affects fish abundance on patch reefs, data are needed on sites with wider distribution of impact levels, ranging from moderate to severe degradation, to compare with less-impacted sites.

In this study we did not investigate natural reef sites due to their inaccessibility to SCUBA. Natural reefs off the coast of the Delmarva peninsula are highly fragmented and sparse, occurring at depths ≥ 27 m. Attempts to locate these by SCUBA diving along commercial trap lines demonstrated that greater amounts of time were needed to locate and sample patch reefs than could be accommodated by no-decompression diving on air or EAN32 gas mix. Natural reefs are commonly targeted by both recreational and commercial fishers; therefore, it is important for future studies to incorporate surveys of natural reefs. Schweitzer, Lipcius & Stevens (2018) surveyed three naturally occurring patch reefs with a remotely operated vehicle in an area targeted by commercial fishers. The mean DI for those sites was 0.37, substantially greater than 0.15 for the study sites in this survey. However, biogenic structure composition and relative fish abundance for those sites or other natural reef sites is unknown.

Our research showed a significant difference in the composition of biogenic structure between sites. Blue mussels were the dominant epifauna at five sites (i.e., ≥22% cover), however they were not observed at the other seven sites. Northern stone coral was observed at ten sites and was dominant (≥17%) at three. Only two sites were not inhabited by sea whips, one of which was an artificial reef constructed ∼6 mo prior to quadrat sampling. Site NH, constructed 2 y prior to being surveyed exhibited <0.01 proportional sea whip coverage, indicating that it takes a minimum of 2 y for sea whips to begin to grow on concrete and metal substrata. However, settlement and growth rates for sea whips (*L, virgulata*) are currently unknown. In contrast, sites BA and NH, both of which were constructed <3 y before surveying, were occupied exclusively by hydroids and mussels, respectively, indicating that those species settle quickly, and are probably replaced over time by longer-lived species such as sea whips and stone corals. We conducted quadrat surveys only once at each site. Repeated quadrat surveys, especially after severe weather events, would give insight on rates of succession, changes in sea whip DI, and sea whip colonization rates on newer artificial reefs.

One limitation of our study is that fish abundance was estimated via two different underwater video survey methods: line transects and non-baited stationary cameras, that were conducted over the course of two years. Ideally, abundance censuses would be conducted in a synoptic fashion; however, weather and water conditions in the Mid-Atlantic Bight were often deemed too hazardous for SCUBA surveys, making it difficult to collect data within specific time blocks. The video surveys acquired from the stationary cameras at four sites (Sites LP, E2, PH, & RG) could result in an upward bias of the MeanCount; nevertheless, fish MeanCount still showed a significant correlation with sea whip abundance at the remaining seven sites. Additional surveys are needed to understand how fish abundance at sites varies seasonally and annually since many of the prominent fish species (e.g., black sea bass and tautog) are seasonal migrators.

**Table 6:** Damage indices for the research sites. Summary of the mean proportional damage for sea whips and the habitat DI by site. *n* is the number of sea whips analyzed at each site. $\overline{x}$ is the mean proportional damage for the measured sea whips. SD is the standard deviation. Max is the highest proportional damage observed. Min is the lowest proportional damage observed. D.I. is the damage index assigned to the site.

Site	*n*	$\overline{\mathbi{x}}$	SD	Max	Min	D.I.	Degradation Category
FW	0	–	–	–	–	–	–
EP	31	0.26	0.19	0.77	0.05	3	Moderate
E2	11	0.02	0.02	0.02	0.00	1	Minimal
PH	19	0.15	0.24	1.00	0.00	2	Minor
BL	17	0.15	0.24	1.00	0.00	2	Minor
RG	19	0.07	0.05	0.18	0.02	2	Minor
MM	24	0.15	0.15	0.47	0.00	2	Minor
SX	21	0.12	0.11	0.40	0.00	2	Minor
NV	26	0.11	0.15	0.66	0.00	2	Minor
NH	7	0.15	0.30	0.82	0.00	2	Minor
BA	0	–	–	–	–	–	–
SW	28	0.15	0.21	0.78	0.00	2	Minor

Our study is the first to quantify the composition of biogenic structure on artificial reefs off the coast of Delmarva peninsula and to show that fish abundance is significantly correlated with the presence and abundance of sea whip corals. Construction of artificial reefs off the coast of Delmarva occurs on an annual basis to increase the local abundance of economically valuable species. Creating artificial reefs near regions with established sea whip coral populations may help facilitate sea whip settlement and colonization of new structures. Future studies to determine variations in fish abundance over time, and to determine the succession of biogenic structure would be useful. In addition, future surveys of naturally occurring patch reefs should be conducted, in order to gain a more detailed assessment of habitat quality in the mesophotic regions of the Mid-Atlantic Bight.

## Conclusion

These results show that there is significant variation in biogenic structure assemblages between artificial reef sites off the coast of the Delmarva peninsula. Fish aggregations and abundance on these artificial reefs are significantly correlated to the abundance of the sea whip coral *L. virgulata*. Sites voided or containing low abundance of *L. virgulata* exhibited the lowest fish abundance. Currently, these artificial reef sites show only minor signs of degradation with no significant difference between sites. It would be important to further these surveys to natural rock reefs and sites that undergo both commercial and recreational fishing pressure to determine if such sites exhibit different levels of sea whip damage, and if higher levels of sea whip degradation effect fish abundance and aggregations. This study could be used as a baseline for current conditions of artificial reef sites off the Delmarva peninsula. Similar surveys should be conducted in the future to monitor succession rates of biogenic structure assemblages. Furthermore, continuing surveys that monitor sea whip settlement rate on new constructed artificial reefs, changes sea whip abundance, and the progression or recovery of damaged sea whips could be valuable to understanding habitat selection and fidelity for economically valuable species in the Mid-Atlantic Bight.

## Additional Information and Declarations

### Data Availability

The following information was supplied regarding data availability: Schweitzer, Cara (2019): SW data_FS.xlsx. figshare. Dataset. https://doi.org/10.6084/m9.figshare.7772951.v1.
